# Fracture Properties of High-Elasticity Asphalt Concrete Reinforced with Rubber Particles and Polyester Fibers

**DOI:** 10.3390/ma19091780

**Published:** 2026-04-27

**Authors:** Jingjiang Wu, Taixu Huo, Juan Wang, Xiaobo Gao, Hui Liu, Jingjing Wang

**Affiliations:** 1 China Construction Seventh Engineering Division Corp. Ltd., Zhengzhou 450004, China; 2 School of Water Conservancy and Transportation, Zhengzhou University, Zhengzhou 450001, China

**Keywords:** high-elasticity asphalt concrete, polyester fibers, rubber particles, fracture properties, aging treatment

## Abstract

**Highlights:**

- The addition of rubber particles increases the fracture energy by approximately 15%.
- The incorporation of polyester fibers increases the fracture energy by approximately 19%.
- Aged asphalt concrete is more susceptible to brittle fracture.
- Aged asphalt concrete exhibits higher fracture toughness.

**Abstract:**

Semi-circular bending tests were conducted on high-elasticity asphalt concrete under different aging conditions to investigate the effects of rubber particles and polyester fiber contents on its fracture properties. Results showed that the incorporation of approximately 3% rubber particles increased the fracture energy by 15%, whereas the addition of 1.2% polyester fibers increased the fracture toughness and fracture energy by 4% and 19%, respectively. Aging-induced oxidative hardening enhanced the overall elastic modulus and interfacial constraint effect of the asphalt mixture, thereby improving the stress transfer efficiency among the rubber particles, polyester fibers, and the surrounding matrix. As a result, both the peak load and fracture toughness increased. However, compared with the unaged state, aged asphalt concrete became more susceptible to brittle fracture, with a decrease in fracture energy and a change in the crack propagation path from a curved to a straight trajectory.

## 1. Introduction

Asphalt concrete is widely used in the construction of roads, bridges, and hydraulic engineering structures. With the continuous economic development and the increasing traffic volume, asphalt concrete pavements have gradually encountered a range of durability issues, including aging and cracking [1,2]. The occurrence of localized cracks in asphalt pavements has become a major factor affecting the long-term service performance of roads [3,4,5].

To enhance the fracture performance of asphalt concrete, retard crack propagation, and optimize mix design, numerous scholars have conducted extensive and in-depth studies focusing on the modification of asphalt concrete. To improve the mechanical properties of asphalt concrete, researchers frequently incorporate materials such as bamboo fiber [6], biochar [7], nanomaterials [8,9,10,11], basalt fiber [12,13], rubber particles [14], and polymer fibers to enhance its crack resistance. Previous studies have demonstrated that incorporating an optimal amount of polyester fibers can improve both the strength and deformation properties of high-elasticity asphalt concrete. Fahad et al. [15] found that, under high loading rate conditions, such as 5 Hz and 60 MPa per second, the indirect tensile strength and permanent deformation resistance of fiber-modified asphalt mixtures were significantly higher than those of unmodified asphalt mixtures. Wang [16] investigated asphalt mixtures containing varying contents of rubber particles and polyester fibers and found that appropriate dosages of these modifiers can effectively improve fracture performance. Saeidi et al. [17] employed the SCB (semi-circular bending) test to evaluate the effect of aging on the low-temperature cracking resistance of asphalt concrete and found that increasing aging significantly reduced the fracture energy and critical energy release rate. Yang [18] and Jin et al. [19] investigated polymer-modified asphalt concrete and reported that it exhibited superior viscoelastic properties and improved low-temperature performance. In addition, the incorporation of synthetic reinforcing fibers (e.g., aramid and polypropylene fibers) has been shown to significantly enhance crack resistance, stiffness, and dynamic modulus of asphalt mixtures [20].

Zhao et al. [21] investigated the effect of rubber particle size on crack initiation and propagation via three-point bending fracture tests combined with Discrete Element Method simulations. The results elucidated the role of rubber particles and showed that concrete modified with fine rubber particles exhibited better performance than concrete modified with coarse rubber particles in restricting microcrack propagation, enhancing mechanical properties, and improving durability. Specifically, the fracture surfaces formed in the Interfacial Transition Zone around larger rubber particles showed higher porosity and roughness coefficients, which significantly decreased the elastic modulus and compressive strength of concrete, especially when rubber particle size was approximately 2 mm [22]. Herráiz et al. [23] prepared Stone Mastic Asphalt modified with polyester fibers and evaluated its performance. The results indicated that polyester fiber-reinforced asphalt concrete exhibited excellent high-temperature stability and water stability. Alnadish et al. [24,25] investigated the properties of synthetic polyester fiber-reinforced steel slag asphalt concrete, revealing that the incorporation of synthetic polyester fibers decreased the elastic modulus while enhancing the tensile strength, rutting resistance, and crack resistance of asphalt concrete. Zarei et al. [26] explored the fracture resistance of asphalt concrete modified with polyester fibers and calcium lignosulfonate. Their findings demonstrated that asphalt specimens with a polyester fiber content of 0.25% and a calcium lignosulfonate content of 6% achieved optimal crack resistance, and the incorporation of both additives effectively mitigated the fracture brittleness of the specimens. Fibers and rubber are among the most commonly used materials for improving asphalt performance [27,28,29,30], and recent studies indicate that fibers are increasingly favored [31]. The effects of rubber particle content, polyester fiber content, and composite aggregate content on the fracture parameters and crack propagation paths of asphalt concrete remain unclear.

Moreover, under repeated heating and cooling cycles, asphalt concrete is susceptible to aging, which impairs its fracture resistance and failure strength. Consequently, asphalt concrete becomes prone to fatigue-induced cracking. Amani et al. [32] and Zieliński [33] analyzed the effects of various aging conditions on the fracture toughness of asphalt mixtures. Fan et al. [34] obtained the fracture energy, fracture toughness, and crack propagation characteristics of asphalt concrete via the SCB test. These findings provide a basis for the durability design of asphalt pavements in cold regions or low-temperature seasons. Ren et al. [35] studied the fracture behavior of asphalt concrete by combining SCB tests with Digital Image Correlation.

In this study, rubber particles and polyester fibers were incorporated into high-elasticity asphalt concrete to improve its mechanical properties. Asphalt concrete mixtures containing different contents of rubber particles and polyester fibers were subjected to various aging treatments. SCB tests were conducted to evaluate the fracture properties of the mixtures and to investigate the effects of rubber particle content, polyester fiber content, and aging duration. In addition, the role of polyester fibers in the aging behavior of asphalt concrete was further examined.

## 2. Experimental Procedures

### 2.1. Raw Materials

In this study, BJ200 asphalt was used as the binder for the bridge expansion joint mixture, and its basic properties are presented in Table 1 in accordance with JTG 3410-2025 [36] and JT/T 798-2019 [37].

The aggregate gradation for asphalt concrete used in seamless expansion joints includes three types: continuous gradation, discontinuous gradation, and single-size (or monomodal) gradation. Single-size aggregates interlock to form a skeletal structure, and without fine aggregates or mineral filler, the voids between aggregates can be filled with asphalt. This enables the asphalt concrete to possess both adequate mechanical properties and deformation capacity. Consequently, asphalt mixtures made with single-size aggregates were selected as the material for seamless expansion joints. A single-size aggregate gradation was adopted, and basalt aggregate with a particle size of 5–10 mm was used.

**Figure 1:**
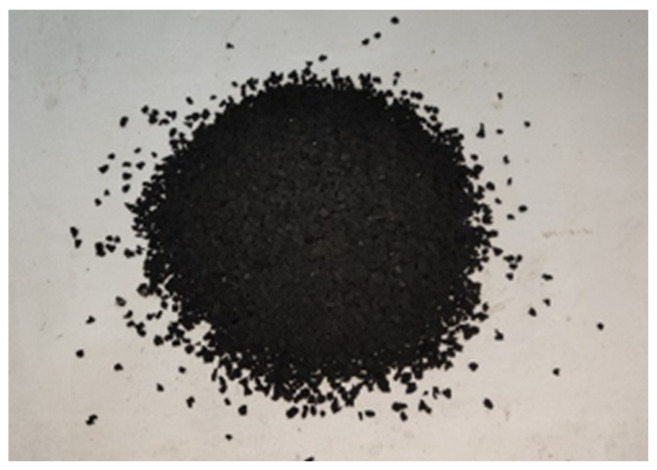
Rubber particles (1~3 mm).

The rubber particles were added at 2%, 3%, and 4% (by mass of aggregate), with a particle size ranging from 1 to 3 mm. The specific parameters for rubber particles are detailed in Table 2, with their schematic diagram shown in Figure 1.

**Figure 2:**
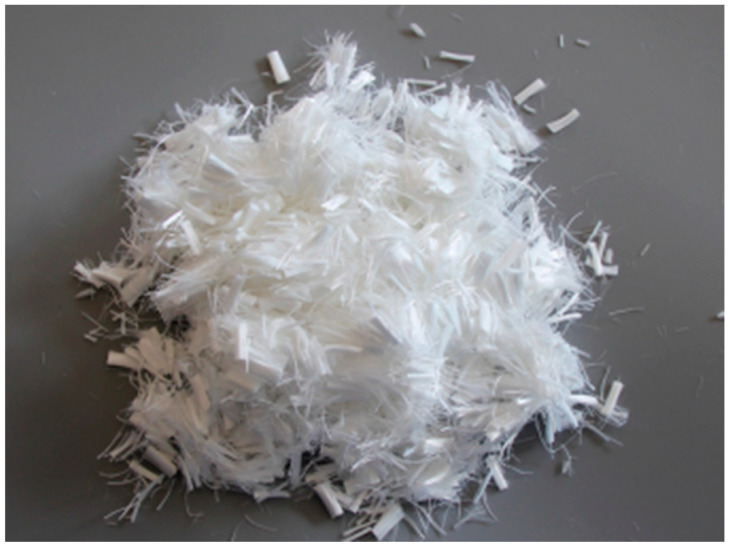
Polyester fiber (Length: 6 mm).

Polyester fibers are incorporated at 0.6%, 1.2%, and 1.8% (by mass of the total mixture), with a fiber length of 6 mm. Specific parameters for the polyester fibers are detailed in Table 3, and their schematic diagram is shown in Figure 2.

High-elasticity asphalt concrete typically requires a significantly higher oil-to-aggregate ratio relative to ordinary asphalt concrete, a factor that compromises its high-temperature stability. Based on existing engineering practice, the asphalt-to-aggregate ratio in seamless expansion joints is typically around 1:5, whereas that in conventional asphalt concrete is generally below 10%. A relatively high asphalt-to-aggregate ratio is required in seamless expansion joints to ensure sufficient elasticity and deformation adaptability [38]. Therefore, when determining the optimum oil-to-aggregate ratio for each mixture, the value corresponding to the highest Marshall stability was selected as the optimal ratio. Based on test results, the optimum oil-to-aggregate ratio was 1:5 when the polyester fiber content was no more than 0.6%, and 1:4 when the polyester fiber content exceeded 0.6%. Consequently, the final mix design for high-elasticity asphalt concrete is presented in Table 4.

### 2.2. Test Method

**Figure 3:**
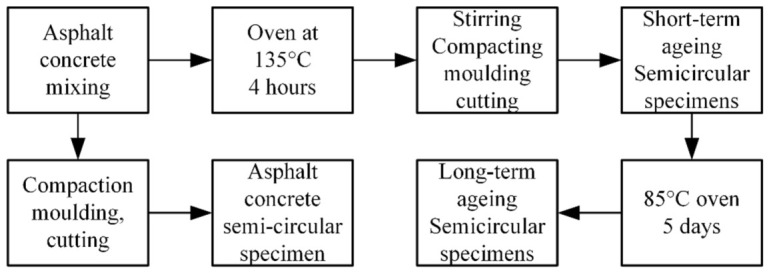
The aging process of high-elasticity asphalt concrete.

This study was conducted according to JTG 3410-2025 [36], and short-term and long-term aging treatments were applied to high-elasticity asphalt concrete to simulate the aging process under different service conditions or durations. The detailed procedures are described as follows (See Figure 3).

(1) Unaged group: SCB fracture specimens of high-elasticity asphalt concrete were prepared directly without aging treatment.
(2) Short-term aging group: Short-term aging tests were performed according to the standard procedure. After mixing, the asphalt mixture was evenly spread on a metal tray and placed in an oven maintained at 135 °C for 4 h. The mixture was turned every hour to ensure sufficient contact with air. After 4 h, the mixture was removed, returned to the mixing drum, and agitated for 45 s. It was then placed into the mold and compacted with 50 blows on each side. After 12 h, the specimen was demolded. The formed specimen was cut into the required semi-circular shape for testing. The short-term aging test primarily simulates the aging conditions experienced during the mixing and paving of asphalt concrete.
(3) Long-term aging group: Before the long-term aging treatment, the asphalt concrete specimens were first subjected to short-term aging according to the same procedure used for the short-term aging group.

After being cut into SCB specimens, the specimens were used for further aging tests. The semi-cylindrical specimens were placed in an iron tray, and the tray was positioned in an oven. The oven temperature was set to 85 °C, and continuous heating under forced ventilation was maintained for 120 h. During this process, the specimens were not touched or moved. After 120 h, the oven was turned off and the door was opened to allow the specimens to cool naturally to room temperature for at least 16 h, after which the loading test was conducted. The long-term aging treatment simulates the performance degradation of asphalt concrete after 5 to 7 years of service.

**Figure 4:**
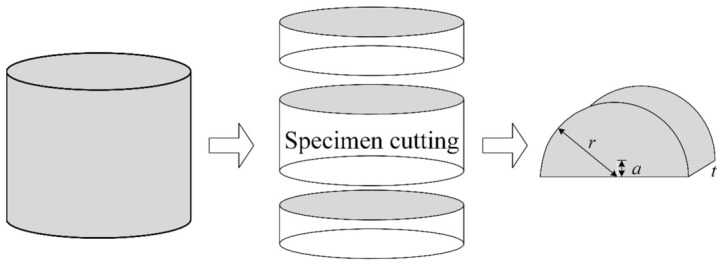
Procedure to form the semi-circular bending sample.

**Figure 5:**
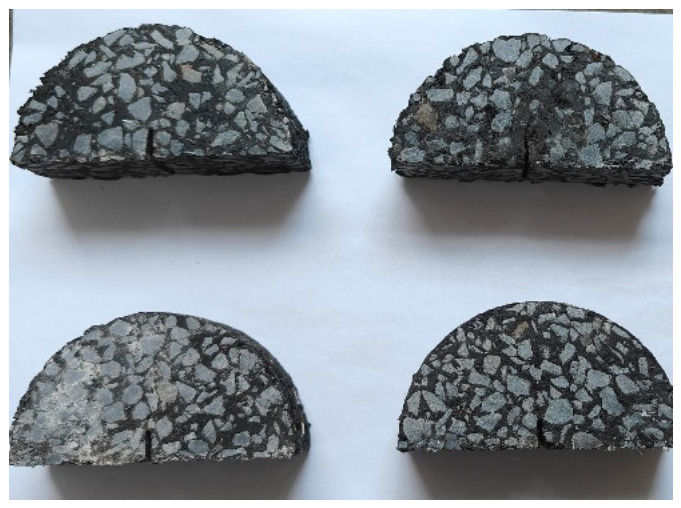
The cut SCB specimen.

The specimens for the SCB test are semi-cylindrical in shape, cut from Marshall specimens. The standard Marshall specimen has the following dimensions: a diameter of 101.6 ± 0.2 mm and a height of 63.5 ± 1.3 mm. The SCB specimens have a diameter of 101.6 mm and a thickness of 30.0 mm. A notch 10 mm long and no more than 1.5 mm wide is cut along the axis of symmetry of the semi-cylindrical specimen to serve as the initial crack. To account for material loss during cutting, the central 30 mm section of the Marshall specimen is used for testing. A single Marshall specimen can yield two semi-cylindrical specimens. The cutting procedure for SCB specimens is illustrated in Figure 4, with the finished specimens shown in Figure 5.

**Figure 6:**
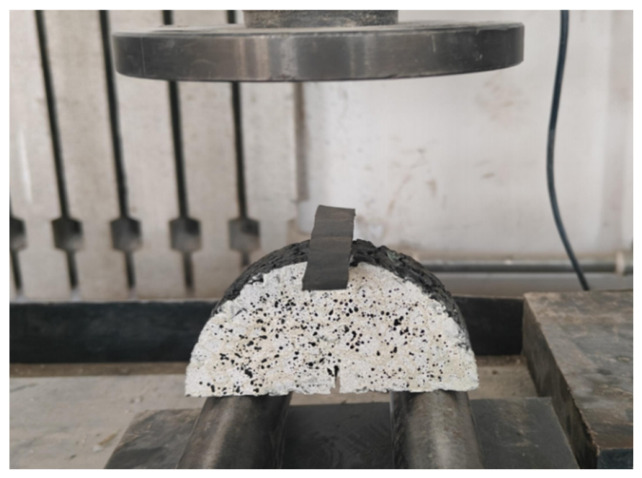
Semi-Circular Bending Fracture test.

The fracture test is performed using a universal testing machine at a temperature of 0 °C, with a support span equal to 0.8 times the specimen diameter. Prior to testing, the specimen is conditioned in an environmental chamber at 0 ± 0.5 °C. The loading rate is maintained at 1 mm/min. During the test, the load and displacement are recorded by the testing machine, and the DIC system is used for observation and image acquisition. The loading process is terminated after specimen failure, and the test data are subsequently saved and analyzed. The fracture test setup is shown in Figure 6.

The fracture parameters of high-elastic asphalt concrete were referenced to the specifications for SCB specimens established by the American Association of Highway and Transportation Engineers (AASHTO), with the fracture energy calculation formula presented in Equation (1).

(1) $$G_{f}=\frac{W_{f}}{A_{lig}}$$

(2) $$W_{f}=\int Pdu$$

(3) $$A_{lig}=(r-a)\times t$$

where: *G*_f_: Fracture energy, J/m^2^; *W*_f_: Fracture work, J; *A*_lig_: Area of the toughness zone, m^2^; *P*: Load, N; *u*: Displacement, m.

The fracture toughness calculation formula is shown in Equation (4).

(4) $$K_{IC}=\frac{P_{max}}{2rt}\sqrt{\pi a}Y$$

(5) $$Y=4.782+1.219\left(\frac{a}{r}\right)+0.063exp(7.045\left(\frac{a}{r}\right))$$

where: *K*_IC_: Fracture toughness, MPa·m^1/2^; *r*: Radius of the specimen, m; *a*: Notch length of the specimen, m; *t*: Thickness of the specimen, m; *P*_max_: Peak load, N; *Y*: Normalized stress intensity factor, non-dimensional (According to the trial design, the parameter values were substituted to obtain Y = 5.284).

## 3. Results and Discussion

### 3.1. Peak Load

Through the aforementioned SCB fracture tests, peak load data for high-elasticity asphalt concrete with different mix proportions and aging periods were obtained and the results are summarized in Table 5.

Based on Equations (1)–(5) and the measured peak load values, the *K*_IC_ and *G*_f_ of the high-elasticity asphalt concrete were calculated (see Table 6 and Table 7).

### 3.2. Effect of Rubber Particle Content on Fracture Properties

Fracture toughness serves as a quantitative measure of a material’s resistance to crack initiation and propagation, reflecting its inherent toughness. As shown in Equation (4), the fracture toughness increases with rising peak load. Fracture energy characterizes the total dissipated energy per unit area during fracture, providing an intuitive representation of a material’s inherent toughness reserve. This metric is crucial for assessing the material’s sustained resistance to crack extension.

**Figure 7:**
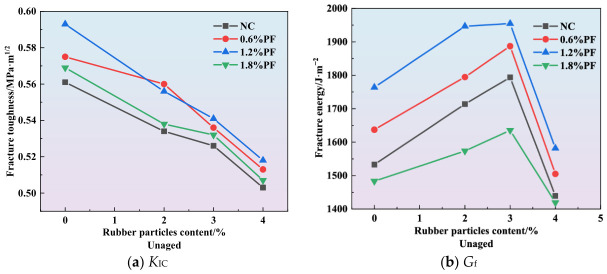
Effect of rubber particles on fracture parameters.

**Figure 8:**
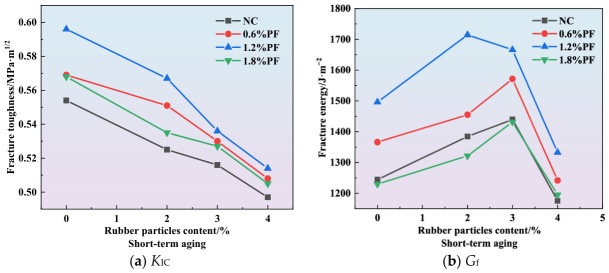
Effect of rubber particles on fracture parameters under short-term aging.

**Figure 9:**
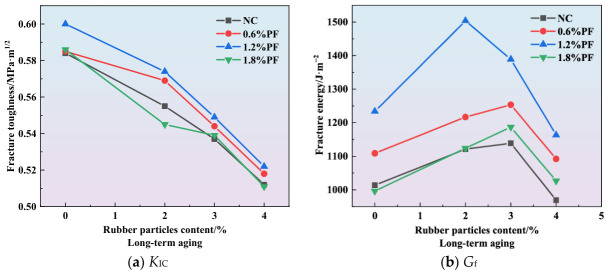
Effect of rubber particles on fracture parameters under long-term aging.

As depicted in Figure 7, Figure 8 and Figure 9, an increase in the rubber particle content results in a gradual decrease in the *K*_IC_ of high-elasticity asphalt concrete. Concurrently, the fracture energy initially increases and subsequently declines sharply. Since fracture toughness depends on specimen dimensions and peak load, it is positively correlated with peak load and shows a similar variation trend for specimens with identical dimensions. As shown in Figure 7b, Figure 8b and Figure 9b, the addition of 3% rubber particles increases the fracture energy by approximately 15%. When both rubber particles and polyester fibers are incorporated into asphalt concrete, the fracture energy initially increases and then decreases with rising rubber particle content. For the unaged group, fracture energy peaks at a rubber particle content of 3%. For specimens from different aging groups, the fracture energy reaches its maximum value when the content of rubber particles is 3%. These findings suggest that, to maximize fracture energy, the rubber particle content should be controlled between 2% and 3%. It is noteworthy that when the content of rubber particles exceeds 4%, the fracture energy reaches its lowest value, falling below that of asphalt concrete without rubber particles.

As shown in Figure 7a, Figure 8a and Figure 9a, the *K*_IC_ of high-elasticity asphalt concrete decreases as the content of rubber particles increases. This phenomenon is primarily due to two key factors. Firstly, the elastic modulus of rubber particles is significantly lower than that of coarse aggregates. Consequently, replacing coarse aggregates with rubber particles reduces the overall stiffness of the mixture. Secondly, when subjected to external loads, rubber particles undergo substantial elastic deformation, dissipating a portion of the applied energy. This energy dissipation reduces the effective stress transmitted to the asphalt matrix and other aggregates. These two factors collectively reduce the peak load of high-elasticity asphalt concrete, and this decline is closely associated with a reduction in the material’s fracture toughness.

As shown in Figure 7b, Figure 8b and Figure 9b, at an optimal content of approximately 3%, rubber particles establish a strong interfacial bond with the asphalt matrix in high-elasticity asphalt concrete when the mixture is subjected to external loading or during crack propagation. As cracks extend, the rubber particles undergo tensile deformation, dissipating a substantial amount of energy, thereby enhancing the material’s fracture energy. However, rubber particles possess a low intrinsic elastic modulus and high deformability, meaning they absorb and dissipate energy during the tensile process until they detach from the asphalt matrix. Thus, the addition of rubber particles at this optimal content effectively enhances the fracture energy of high-elasticity asphalt concrete. Owing to the large specific surface area of rubber particles, asphalt cannot fully wet them when their content exceeds 3%. If the asphalt content is not increased proportionally, the internal structure of the mixture becomes loose, and porosity increases. This significantly compromises the energy dissipation efficiency during crack propagation, ultimately resulting in a decrease in fracture energy.

### 3.3. Effect of Polyester Fiber Content on Fracture Properties

**Figure 10:**
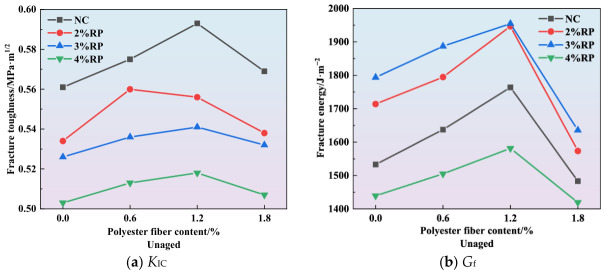
Effect of polyester fiber on fracture parameters.

**Figure 11:**
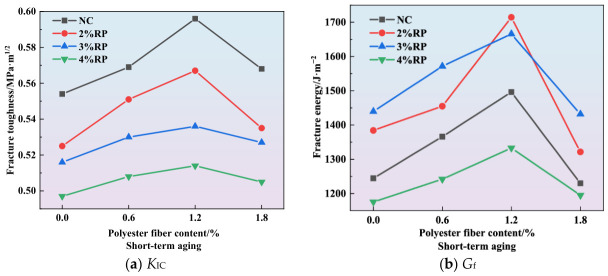
Effect of polyester fiber on fracture parameters under short-term aging.

**Figure 12:**
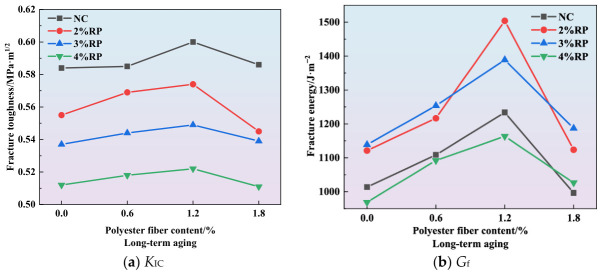
Effect of polyester fiber on fracture parameters under long-term aging.

The curves showing the effect of polyester fiber content on fracture toughness and fracture energy are presented in Figure 10, Figure 11 and Figure 12. As illustrated in Figure 10a, Figure 11a and Figure 12a, the incorporation of polyester fibers is capable of improving the fracture toughness of high-elasticity asphalt concrete. As a polymeric fiber, polyester has significantly lower rigidity than inorganic rigid fibers, and primarily functions to inhibit microcracking within the asphalt matrix. At a content of approximately 1.2%, polyester fibers can disperse uniformly throughout the asphalt matrix, thereby promoting the formation of a three-dimensional network structure. When microcracks initiate within the mixture, the polyester fibers can transfer a portion of the stress, alleviating stress concentration at the crack tips and thereby enhancing *K*_IC_. However, excessive polyester fibers tend to agglomerate due to poor dispersion. Under external loading, cracks are more likely to initiate within these agglomerated zones, which results in reduced overall strength and fracture toughness of the specimens.

As illustrated in Figure 10b, Figure 11b and Figure 12b, the incorporation of polyester fibers is capable of significantly improving the *G*_f_ of high-elasticity asphalt concrete. At the optimal content of 1.2%, polyester fibers can fully exert their reinforcing and toughening effects. When cracks propagate to the fiber positions, the load is transferred to the fibers, and their anchoring effect inhibits further crack propagation. As external loads continue to increase, cracks propagate at the fiber-asphalt matrix interface, resulting in debonding at the fiber-asphalt interface or fiber fracture. The intrinsic high tensile strength of the polyester fiber enhances the material’s fracture energy. Moreover, for existing cracks, the bridging effect of fibers prevents the high-elasticity asphalt concrete from experiencing direct and unstable fracture, thereby improving its ductility.

### 3.4. Effect of Different Aging Durations on Fracture Properties

#### 3.4.1. Fracture Toughness

**Figure 13:**
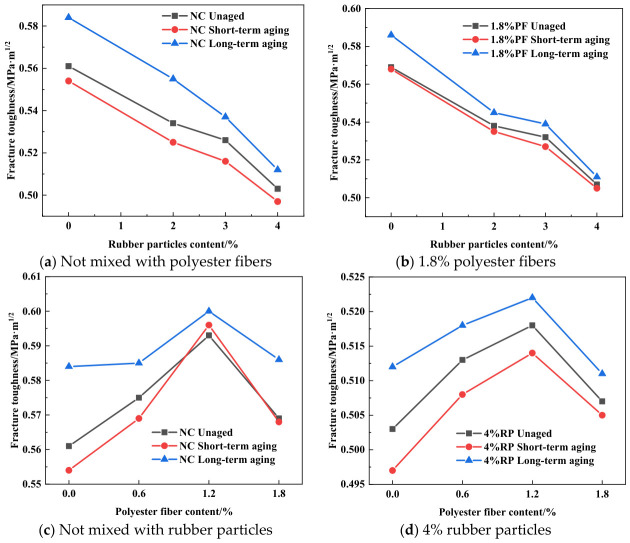
Fracture toughness under different aging durations.

As illustrated in Figure 13, following short-term aging treatment, the fracture toughness of high-elasticity asphalt concrete shows a slight decrease compared with the unaged specimens. At this stage, although short-term aging impairs the rheological properties of the asphalt mixture, the inherent favorable characteristics of the asphalt matrix mitigate performance degradation, resulting in only a marginal decrease in fracture toughness.

**Figure 14:**
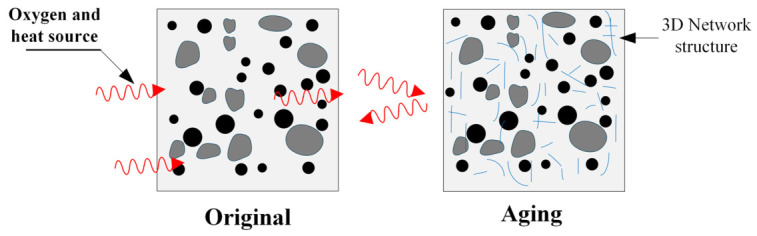
Schematic diagram of the blocking effect of the three-dimensional polyester fiber network on heat, oxygen, and moisture ingress.

Furthermore, increasing the contents of polyester fibers and rubber particles mitigates the detrimental effect of aging on fracture toughness. The unique network-like distribution of polyester fibers not only delays the aging process of high-elasticity asphalt concrete but also slows the rate of decline in its fracture toughness. Specifically, as illustrated in Figure 14, the mesh-like distribution of polyester fibers provides a degree of protection for high-elasticity asphalt concrete [38,39]. Concurrently, the interfacial bonding between polyester fibers and the asphalt matrix maintains effective load transfer capacity during the early aging stages. This compensates for the loss of inherent toughness in the asphalt matrix while mitigating the detrimental effects of aging on fracture toughness.

**Figure 15:**
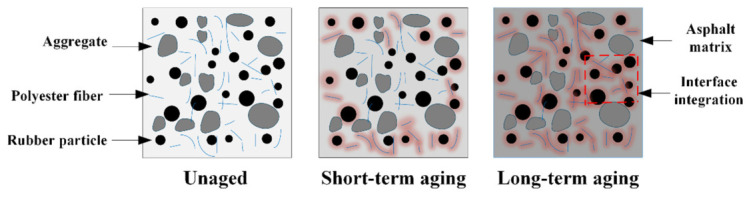
Schematic diagram illustrating the interface integration after asphalt aging.

As illustrated in Figure 15, during long-term aging, the rubber particles undergo progressive melting and interfacial modification, leading to the formation of a stable compatible phase with the asphalt matrix. This phase transformation enhances the stiffness of the rubber particles, consequently improving the mixture’s resistance to external loading, which is manifested as an increase in peak load. Since fracture toughness is positively correlated with peak load, the *K*_IC_ of high-elasticity asphalt concrete also shows an increasing trend under long-term aging treatment. The above research conclusions are consistent with those reported in reference [40,41].

#### 3.4.2. Fracture Energy

**Figure 16:**
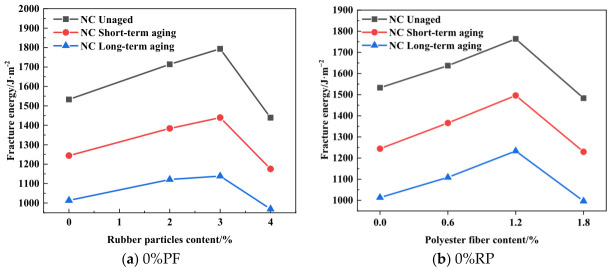
Fracture energy results under different aging times.

As illustrated in Figure 16, the fracture energy of high-elasticity asphalt concrete exhibits the following order across different aging treatments: unaged group > short-term aging group > long-term aging group. As shown in Figure 16a, the fracture energy of the short-term aging group and long-term aging group decreased by 19.7% and 36.5%, respectively, compared with that of the unaged group. Similarly, Figure 16b shows that the fracture energy of the short-term aging group and long-term aging group decreased by 15.2% and 30.1%, respectively, compared with that of the unaged group. Comparative analysis indicates that the mixture containing only polyester fibers exhibits better resistance to aging.

It is clear that prolonged aging treatment results in higher *P*_max_ and *K*_IC_ values of high-elasticity asphalt concrete compared to the unaged material. Nevertheless, the increased brittleness associated with aging gives rise to a decline in fracture energy. After long-term aging, the high-elasticity asphalt concrete exhibits reduced fracture energy and diminished energy dissipation capacity during the fracture process, making it more prone to brittle fracture without noticeable plastic deformation.

The differences between fracture energy and fracture toughness after aging primarily stem from their distinct failure stages. Fracture toughness, which is mainly governed by the peak load, reflects crack initiation resistance, whereas fracture energy, measured by the area under the complete load–displacement curve, characterizes deformation capacity and energy dissipation during crack propagation. Aging increases the stiffness and peak load-carrying capacity of the asphalt mixture, but also enhances its brittleness, resulting in increased fracture toughness and reduced fracture energy. For polyester fiber-modified mixtures, the bridging and anchoring effects of the fibers improve the load-carrying capacity during crack initiation. However, aging-induced matrix embrittlement and the reduced interfacial energy dissipation capacity weaken the dissipation of tensile and debonding energy during crack propagation, thereby leading to a decrease in fracture energy. This finding is consistent with that reported in Ref. [42].

#### 3.4.3. Effect of Aging Duration on Crack Propagation Pathways

**Figure 17:**
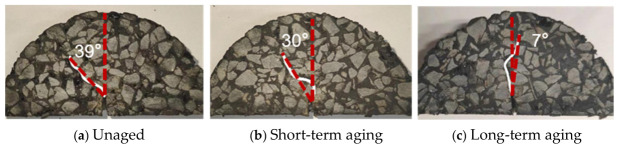
Crack propagation paths and crack opening angles under different aging durations.

Figure 17 shows the crack propagation paths and crack opening angles corresponding to different aging durations.

High-elasticity asphalt concrete exhibits significant differences in crack opening angles under varying aging durations: the crack opening angles of unaged and short-term aging specimens are markedly greater than those of long-term aged ones. Specifically, unaged and short-term aging asphalt retain favorable viscoelastic properties and toughness, exhibiting strong interfacial bonding with aggregates and rubber particles. During crack propagation, energy is dissipated significantly due to the need to overcome both the viscous deformation resistance of the asphalt and the resistance from the interfacial bonding. To fully dissipate energy, cracks propagate at a large opening angle in a meandering pattern, as illustrated in Figure 17a,b, thereby extending the propagation path length and enhancing energy dissipation efficiency. Conversely, as aging progresses, the viscoelasticity and toughness of the asphalt binder deteriorate significantly, and the interfacial bonding strength weakens. At this stage, the crack opening angle significantly decreases, as illustrated in Figure 17c, and the cracks propagate rapidly along a straight path with lower energy dissipation efficiency.

## 4. Conclusions

In order to investigate the effects of rubber particles and polyester fibers on the fracture behavior of high-elasticity asphalt concrete, SCB fracture tests were conducted, and the mechanisms by which these modifiers enhance fracture performance were analyzed. Furthermore, short-term and long-term aging were simulated to mimic different service durations, enabling an examination of the fracture behavior of high-elasticity asphalt concrete under varying service periods. The role of polyester fibers in the mitigation of the aging-induced degradation of asphalt concrete was also elucidated. Based on the above results, the following conclusions are drawn:

1. As the rubber particle content increases, the fracture toughness and peak load of high-elasticity asphalt concrete gradually decrease, while the fracture energy initially increases before decreasing. At a rubber particle content of 3%, the fracture energy of high-elasticity asphalt concrete increases by approximately 15% compared with the group without rubber particles. However, when the rubber particle content exceeds 4%, the asphalt binder cannot fully encapsulate all rubber particles, which may result in an increase in internal defects and a rapid reduction in fracture energy.
2. As the polyester fiber content increases, the fracture toughness and fracture energy of high-elasticity asphalt concrete tend to increase first and then decrease. When the polyester fiber content is 1.2%, the fracture energy reaches its maximum value, increasing by 15.1%, 20.2%, and 21.7% in the three aging groups, respectively. The incorporation of polyester fibers promotes the formation of a three-dimensional network structure within asphalt concrete, enhancing its crack bridging capacity.
3. High-elasticity asphalt concrete exhibits higher fracture toughness under long-term service conditions than both the unaged group and the short-term aged group. When rubber particles are incorporated at 4%, the fracture toughness of the short-term aged group tends to decrease. However, the fracture energy gradually decreases with increasing service duration. The maximum fracture energy is achieved at 3% rubber particle content or 1.2% polyester fiber content under single-additive conditions.
4. Unaged and short-term aged asphalt concrete exhibits favorable elastoplastic behavior, with cracks opening at an angle of 39 degrees and propagating along paths with higher resistance. In contrast, after long-term aging, the asphalt concrete becomes more brittle and less viscoelastic, causing cracks to open at smaller angles and propagate rapidly along paths with lower resistance.

## Figures and Tables

**Table 1:** Basic performance indicators of BJ200 asphalt provided by Prismo, Lancashire, UK [16].

Parameters	BJ200
Softening point, °C	93.8
Penetration (5 °C), cm	14.5
Penetration (25 °C), 0.1 mm	25
Elastic recovery rate, %	75
Viscosity (190 °C), Pa·s	4.5
70 °C Complex modulus, kPa	6.0
70 °C Phase angle, °	57
70 °C Non-recoverable creep compliance Jnr3.2, kPa^−1^	0.07
70 °C Percent recovery R3.2, %	80

**Table 2:** Specific parameters for rubber particles (The rubber particles were produced by Dujiangyan Xinhua Yi Rubber Products Business Department, Dujiangyan, China) [16].

Indicators	Combustion Residues (%)	Ash Content (%)	Rubber Content (%)	Fiber Content (%)	Moisture Content (%)
Value	37.5	4.5	51	0.5	0.6

**Table 3:** Specific parameters of polyester fiber.

Indicators	Specific Gravity	Modulus of Elasticity (GPa)	Elongation at Break (%)	Tensile Strength (MPa)	Melting Point (°C)	Ignition Point (°C)
Value	0.91	13.5	9 ± 3	550	259	554

**Table 4:** Mix ratio of high-elasticity asphalt concrete.

Specimen Number	Number of Each Group	Oil-Stone Ratio	Asphalt kg/m^3^	Aggregate kg/m^3^	Rubber Particles kg/m^3^	Rubber Particles%	Polyester %
NC	3	1:5	388	1938	0	0	0
F0.6		1:5	388	1938	0	0	0.6
F1.2		1:4	451	1803	0	0	1.2
F1.8		1:4	451	1803	0	0	1.8
R2		1:5	388	1899	39	2	0
R3		1:5	388	1880	58	3	0
R4		1:5	388	1860	78	4	0
R2F0.6		1:5	388	1899	39	2	0.6
R2F1.2		1:4	451	1767	36	2	1.2
R2F1.8		1:4	451	1767	36	2	1.8
R3F0.6		1:5	388	1880	58	3	0.6
R3F1.2		1:4	451	1749	54	3	1.2
R3F1.8		1:4	451	1749	54	3	1.8
R4F0.6		1:5	388	1860	78	4	0.6
R4F1.2		1:4	451	1731	72	4	1.2
R4F1.8		1:4	451	1731	72	4	1.8

Note: (1) NC denotes the conventional group; R2F0.6 represents high-elasticity asphalt concrete with 2% rubber particles and 0.6% polyester fibers. (2) The rubber particle content is defined as the mass ratio of rubber particles to aggregate, whereas the polyester fiber content is defined as the mass ratio of polyester fibers to the total mass of asphalt concrete.

**Table 5:** Peak load in the high-elasticity asphalt concrete SCB fracture test.

Specimen Number	Peak Load/(Mean ± SD, N)					
	Unaged	CV, %	Short-Term Aging	CV, %	Long-Term Aging	CV, %
NC	1797.14 ± 106.53	5.9	1774.31 ± 132.25	7.5	1872.18 ± 110.98	5.9
F0.6	1841.53 ± 137.26	7.4	1821.54 ± 107.98	5.9	1874.51 ± 139.73	7.4
F1.2	1899.54 ± 143.61	7.5	1908.81 ± 144.32	7.7	1921.66 ± 145.28	7.5
F1.8	1821.36 ± 122.52	6.7	1819.34 ± 122.38	6.7	1877.85 ± 126.32	6.7
R2	1711.32 ± 101.95	5.9	1681.48 ± 100.17	6.0	1777.14 ± 105.87	5.9
R2F0.6	1794.65 ± 106.38	5.9	1764.94 ± 140.09	7.9	1822.94 ± 108.06	5.9
R2F1.2	1780.51 ± 141.33	7.9	1817.14 ± 135.44	7.5	1837.15 ± 145.82	7.9
R2F1.8	1723.84 ± 59.29	3.4	1715.31 ± 59.00	3.4	1745.63 ± 60.04	3.4
R3	1684.85 ± 133.73	7.9	1654.31 ± 131.31	7.9	1719.45 ± 136.48	7.9
R3F0.6	1717.42 ± 115.53	6.7	1697.54 ± 114.19	6.7	1741.36 ± 117.14	6.7
R3F1.2	1733.65 ± 129.22	7.4	1717.46 ± 136.32	7.9	1757.91 ± 131.03	7.4
R3F1.8	1703.14 ± 101.46	5.9	1688.56 ± 100.59	6.0	1725.16 ± 102.78	5.9
R4	1612.62 ± 121.93	7.5	1592.18 ± 120.38	7.6	1641.31 ± 124.09	7.5
R4F0.6	1642.05 ± 56.48	3.4	1627.94 ± 96.50	5.9	1659.64 ± 57.09	3.4
R4F1.2	1658.65 ± 131.65	7.9	1646.35 ± 56.63	3.4	1671.18 ± 132.65	7.9
R4F1.8	1624.31 ± 96.29	5.9	1617.61 ± 120.57	7.5	1637.56 ± 218.02	13.3

**Table 6:** Fracture toughness of asphalt concrete with different degrees of aging.

Specimen Number	*K*_IC_/(Mean ± SD, MPa·m^1/2)^					
	Unaged	CV, %	Short-Term Aging	CV, %	Long-Term Aging	CV, %
NC	0.561 ± 0.033	5.8	0.554 ± 0.041	7.4	0.584 ± 0.035	5.9
F0.6	0.575 ± 0.043	7.4	0.568 ± 0.034	5.9	0.585 ± 0.044	7.4
F1.2	0.593 ± 0.045	7.5	0.596 ± 0.045	7.5	0.600 ± 0.046	7.6
F1.8	0.568 ± 0.038	6.7	0.568 ± 0.038	6.7	0.586 ± 0.039	6.7
R2	0.534 ± 0.032	5.9	0.525 ± 0.031	6.0	0.555 ± 0.033	5.9
R2F0.6	0.560 ± 0.034	6.0	0.551 ± 0.044	8.0	0.569 ± 0.034	5.9
R2F1.2	0.556 ± 0.044	7.9	0.567 ± 0.042	7.4	0.574 ± 0.045	7.9
R2F1.8	0.538 ± 0.018	3.4	0.535 ± 0.018	3.4	0.545 ± 0.018	3.4
R3	0.526 ± 0.042	7.9	0.516 ± 0.041	8.0	0.537 ± 0.042	7.9
R3F0.6	0.536 ± 0.036	6.6	0.530 ± 0.036	6.8	0.544 ± 0.036	6.7
R3F1.2	0.541 ± 0.040	7.4	0.536 ± 0.042	7.9	0.549 ± 0.041	7.4
R3F1.8	0.532 ± 0.032	5.9	0.527 ± 0.031	6.0	0.538 ± 0.032	6.0
R4	0.504 ± 0.038	7.6	0.497 ± 0.038	7.6	0.512 ± 0.039	7.6
R4F0.6	0.513 ± 0.017	3.4	0.508 ± 0.030	5.9	0.518 ± 0.018	3.5
R4F1.2	0.518 ± 0.041	8.0	0.514 ± 0.018	3.4	0.522 ± 0.041	7.9
R4F1.8	0.507 ± 0.030	5.9	0.505 ± 0.037	7.4	0.511 ± 0.068	13.4

**Table 7:** Fracture energy of asphalt concrete with different degrees of aging.

Specimen Number	*G*_f_/(Mean ± SD, J·m^−2^)					
	Unaged	CV, %	Short-Term Aging	CV, %	Long-Term Aging	CV, %
NC	1532.86 ± 65.92	4.3	1244.29 ± 60.97	4.9	1013.54 ± 58.79	5.8
F0.6	1637.15 ± 178.45	10.9	1365.91 ± 128.39	9.4	1108.65 ± 106.43	9.6
F1.2	1763.66 ± 142.85	8.1	1495.99 ± 160.07	10.7	1233.64 ± 56.75	4.6
F1.8	1483.24 ± 94.93	6.4	1229.61 ± 75.01	6.1	996.34 ± 107.60	10.8
R2	1713.79 ± 89.11	5.2	1384.11 ± 76.13	5.5	1121.19 ± 69.51	6.2
R2F0.6	1794.59 ± 202.78	11.3	1454.94 ± 120.76	8.3	1216.63 ± 103.41	8.5
R2F1.2	1946.21 ± 147.91	7.6	1714.31 ± 195.43	11.4	1503.63 ± 106.75	7.1
R2F1.8	1573.54 ± 143.19	9.1	1321.35 ± 92.50	7.0	1123.65 ± 124.72	11.1
R3	1793.49 ± 86.09	4.8	1439.51 ± 64.78	4.5	1138.56 ± 56.93	5.0
R3F0.6	1886.94 ± 192.47	10.2	1571.46 ± 155.58	9.9	1253.32 ± 112.81	9.0
R3F1.2	1954.39 ± 170.04	8.7	1665.83 ± 146.59	8.8	1388.69 ± 94.43	6.8
R3F1.8	1635.47 ± 112.85	6.9	1431.61 ± 94.49	6.6	1186.84 ± 122.25	10.3
R4	1439.47 ± 82.05	5.7	1175.41 ± 59.95	5.1	968.94 ± 42.64	4.4
R4F0.6	1504.94 ± 165.54	11.0	1241.65 ± 130.37	10.5	1091.96 ± 88.45	8.1
R4F1.2	1581.51 ± 115.45	7.3	1332.96 ± 102.64	7.7	1163.36 ± 61.66	5.3
R4F1.8	1419.36 ± 139.10	9.8	1194.96 ± 109.93	9.2	1026.32 ± 99.56	9.7

## Data Availability

The raw/processed data required to reproduce these findings cannot be shared at this time as the data also forms part of an ongoing study. Further inquiries can be directed to the corresponding authors.
